# Transcription Factors ClrB and XlnR and Their Effect on the Transcription of Cellulase Genes in the Filamentous Fungus *Penicillium verruculosum*

**DOI:** 10.3390/ijms252413373

**Published:** 2024-12-13

**Authors:** Andrey Chulkin, Valeriy Kislitsin, Igor Sinelnikov, Arkady Sinitsyn, Ivan Zorov, Pavel Volkov, Aleksandra Rozhkova

**Affiliations:** 1 Federal Research Centre “Fundamentals of Biotechnology”, Russian Academy of Sciences, Moscow 119071, Russiaapsinitsyn@gmail.com (A.S.); amrojkova@yahoo.com (A.R.); 2 M. Aitkhozhin Institute of Molecular Biology and Biochemistry, Almaty 050012, Kazakhstan; 3 Chemistry Department, M.V. Lomonosov Moscow State University, Moscow 119991, Russia

**Keywords:** transcription factors, *Penicillium verruculosum*, cellulases, induction, qPCR, transcriptome

## Abstract

The filamentous fungus *Penicillium verruculosum* (anamorph *Talaromyces verruculosus*) has been shown to be an efficient producer of secreted cellulases, used in biorefinery processes. Understanding the mechanisms of regulation of cellulase gene expression in the fungus *P. verruculosum* is a current task in industrial biotechnology, since it allows for targeted changes in the composition of the complex secreted by the fungus. Expression of cellulase genes in fungi is regulated mainly at the level of transcription via pathway-specific transcription factors (TF), the majority of which belong to the Zn(II)2Cys6 family of zinc binuclear cluster proteins. Transcriptional regulation of cellulase genes may have a species-specific pattern and involves several transcription factors. In this study, we used a qPCR method and transcriptome analysis to investigate the effect of knockouts and constitutive expression of genes encoding homologues of the regulatory factors XlnR and ClrB from *P. verruculosum* on the transcription of *cbh1*, *egl2*, and *bgl1* genes, encoding three key cellulases, cellobiohydrolase, endoglucanase, and β-glucosidase, in the presence of various inducers. We have shown that the transcription factor XlnR of the filamentous fungus *P. verruculosum* is strictly responsible for the transcription of the main cellulolytic genes (*cbh1*, *egl2*, and *bgl1*) in the presence of xylose and xylobiose, but not in the presence of cellobiose. ClrB/Clr-2, a homologue from *P. verruculosum*, does not represent the main transcription factor regulating transcription of cellulolytic genes in the presence of selected inducers, unlike in the cases of *Aspergillus nidulans*, *Aspergillus niger*, and *Penicillium oxalicum;* apparently, it has a different function in fungi from the genus *Talaromyces*. We have also shown that constitutive expression of the transcription factor XlnR resulted in 3.5- and 2-fold increases in the activity of xylanase and β-glucosidase in a B1-XlnR enzyme preparation, respectively. In a practical sense, the obtained result can be used for the production of enzyme preparations based on the *P. verruculosum* B1-XlnR strain used for the bioconversion of renewable cellulose-containing raw materials into technical sugars.

## 1. Introduction

The filamentous fungus *Penicillium verruculosum* (anamorph *Talaromyces verruculosus*) has been shown to be an efficient producer of a cellulase complex [1]. Cellulases are glycoside hydrolases (GHs) that efficiently depolymerize cellulose into its building blocks, sugar monomers, and are commonly used in biomass degradation [2]. Complete degradation of the cellulose in renewable cellulose-containing raw materials requires a synergistic action of three different types of cellulases: endo-β-1,4-glucanases, cellobiohydrolases, and β-glucosidases [3]. The *P. verruculosum* strain is able to secrete up to 50–60 g/L of protein, 80% of which is composed of cellulases. Among them, cellobiohydrolase CBH1 (GH family 7), endoglucanase EGL2 (GH family 5), and β-glucosidase BGL1 (GH family 3) account for 36–41%, 12–18%, and 3–5%, respectively [4].

Mechanisms of cellulase and (hemi)cellulase gene expression have been studied in detail in several fungal species, including *Aspergillus* spp. [5,6], *Trichoderma* spp. [7], *Neurospora crassa* [8], and others [9,10]. The expression of secreted hydrolases is regulated mainly at the level of transcription via pathway-specific transcription factors (TFs), the majority of which belong to the Zn(II)2Cys6 family of zinc binuclear cluster proteins [11]. The pattern of transcriptional regulation of cellulase genes may be species-specific and involve several transcription factors. For example, transcriptional activator Xyr1 in the filamentous fungus *Trichoderma reesei* and its homologue XlnR from *Aspergillus niger* and *Penicillium oxalicum* are responsible for the expression of both cellulases and hemicellulases, whereas in *N. crassa* and *Aspergillus nidulans*, they regulate only hemicellulases [12]. Conversely, the transcription of cellulase genes in *N. crassa* is under the control of two activators, Clr-1 and Clr-2. In the case of *A. nidulans* and *A. niger*, this function is performed by their homologues, ClrA and ClrB. In *N. crassa*, Clr-1 activates the transcription of a number of genes required for cellulose utilization and also controls the expression of Clr-2, the major activator of cellulases. In *A. nidulans* and *A. niger*, ClrA plays a role in cellulase induction but is not required for *clrB* transcription [13]. These regulators become activated in the presence of inducers, which usually are by-products of polysaccharide degradation [14]. Inducing compounds may vary among fungal species. It was shown that (hemi)cellulase genes in *A. niger* are induced by D-xylose [15], whereas in *Penicillium* spp., *A. nidulans*, and *T. reesei*, induction occurs in the presence of gentiobiose, cellobiose, or sophorose, respectively [16,17,18]. Earlier, we investigated the effect of different inducers on *cbh1* transcription in the filamentous fungus *P. verruculosum*. We showed that sophorose, D-xylose, short xylooligosaccharides, gentiobiose, and cellobiose could act as direct inducers of the *cbh1* gene [19]. It is evident that the expression of fungal cellulases is controlled by a network of diverse transcription factors and associated inducers [20].

The present work aimed to study the role of homologues of the regulatory factors Xyr-1/XlnR and Clr-2/ClrB from *P. verruculosum* in the transcription of the *cbh1*, *egl2*, and *bgl1* genes, encoding cellulases CBH1, EGL2, and BGL1, respectively, as well as to evaluate the change in the profile of cellulases/hemicellulases secreted by knockout strains and strains overexpressing the corresponding TFs.

Understanding transcriptional interactions in a particular fungal strain, including *P. verruculosum*, will enable the control of the biosynthesis process of target cellulases/hemicellulases, and new strains with improved enzymatic activities can be obtained.

## 2. Results

### 2.1. Identification and Cloning of P. verruculosum TF Genes

The amino acid sequences of TFs XlnR, Clr-1, and Clr-2 from *N. crassa* (strain OR74A, GeneBank ID: XP_962611.1, XP_011394265.1, and XP_962712.2) and XlnR, ClrA, and ClrB from *A. nidulans* (strain FGSC A4, GeneBank ID: XP_663412.1, XP_660973.1, and XP_680879.1) were compared with *P. verruculosum* genome data using the tblastn program from the BLAST+ package (version 2.12.0) [21], with an e-value cutoff of -50. The data are presented in Figure S1.

In the *P. verruculosum* genome, one homologue of the Clr-2/ClrB TFs, named ClrB, was found, and two homologues were found for the TF XlnR. The polynucleotide sequence with the highest homology was named the *xlnR* gene and selected for further analysis. No homologues of TF Clr-1 and ClrA were found with the selected parameters.

**Figure 1:**
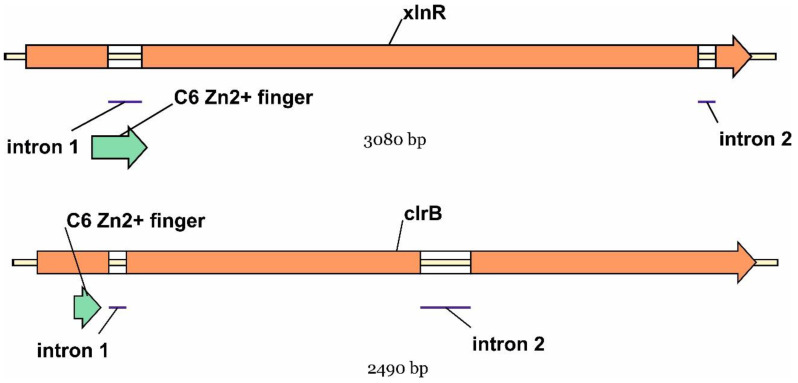
Schemes of *clrB* and *xlnR* genes of *P. verruculosum*. The exon–intron structure and zinc finger coding regions (green arrows) are indicated.

Thus, the complete sequences of the coding regions of *clrB* and *xlnR* genes from *P. verruculosum* were obtained. The maps of these genes are shown in Figure 1. Nucleotide sequences of the coding regions and derived amino acid sequences are presented in Figure S2.

### 2.2. Knockout of TF Genes

Earlier, we adapted a CRISPR/Cas9-based method of genome editing for gene manipulations in the *P. verruculosum* strain B1-221-6. The sequencing of the PCR-amplified fragment of the target gene *cbh1* showed that about 50% of clones had a frameshift-type mutation in the protospacer action region [22]. In this study, this technique was applied to knock out *clrB* and *xlnR* genes.

Using ChopChop software version 3.0.0 (https://chopchop.cbu.uib.no/, accessed on 7 December 2024), we selected spacers for the *xlnR* and *clrB* genes of *P. verruculosum* in the 5′ region of coding parts, located upstream of the Zn-finger domain:

*clrB*: 5’- CTACAACAAGACGAAGCGAG -3’

*xlnR*: 5’- GCATTCTGCTGATACGGTTG -3’

Then, the plasmids were created to knock out these genes (Figure S3), as described in Section 3.3.

*P. verruculosum* strain B1-221-6 was co-transformed with the resulting plasmids, which were combined with the pSgRNAniaD plasmid. After selecting clones, DNA was isolated from the mycelium of 10 transformants for each TF. Fragments of edited genes containing the protospacer sequence were amplified and sequenced. The frequency of frameshift mutations for the *xlnR* and *clrB* genes was about 10% and 50%, respectively.

**Figure 2:**
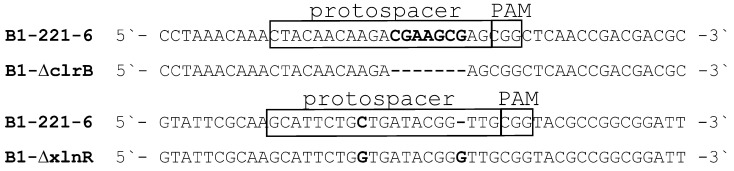
Sequence alignment of protospacer regions between the B1-221-6 strain and the selected knockout strains B1-ΔclrB and B1-ΔxlnR. Protospacers and PAMs (protospacer adjacent motifs) are indicated. Nucleotide substitutions and deletions are shown in bold.

For each TF, one transformant with a frameshift mutation was selected for further analysis (Figure 2). The knocked-out recombinant strains were named B1-ΔclrB and B1-ΔxlnR.

### 2.3. Overexpression of TF Genes

Earlier, the efficiency of the promoter of the *gpdA* gene from *P. verruculosum* was tested by fusing it with the coding part of the β-glucosidase *bgl1* gene from *A. niger* and with the transcription terminator of the *cbh1* gene from *P. verruculosum*. During growth under CCR conditions on a glucose-containing medium, a unique (single) major band corresponding to the β-glucosidase of *A. niger* was observed in the SDS-PAGE of the culture fluid. The MALDI-TOF analysis confirmed that the band represented β-glucosidase [23].

The coding regions of the cloned TFs were fused with the constitutive promoter of the *gpdA* gene and with the transcription terminator of the *cbh1* gene from *P. verruculosum* using a Gibson Assembly^®^ Master Mix (NEB). The procedure was performed by replacing the *cas9* gene in the pGpdCas9 plasmid [22] with the coding regions of the TF genes. As a result, pGpdXlnR and pGpdClrB plasmids were obtained (Figure S4).

The plasmids were introduced into the auxotrophic strain B1-537 by co-transformation [24] with the pSTA-10 plasmid [25], which carried the *niaD* gene from *A. niger*. For each TF, DNA from 15 transformants was used for the qPCR-based selection of strains carrying plasmids with TF genes under the control of the *gpdA* promoter from *P. verruculosum*. For each TF, one transformant with an apparently high copy number of plasmids integrated into the genome was selected for further analysis. The recombinant strains were named B1-clrB and B1-xlnR.

### 2.4. Characterization of the Transcription of cbh1, bgl1, and egl2 Genes Under Different Inducers in P. verruculosum Strains via qPCR

Mycelia grown on complete medium (CM) were transferred into test tubes with MM supplemented with a Ci-PO_4_ buffer as a derepressive carbon source in order to turn off the CCR mechanism. After a 1 h incubation period, the inducer was added. The following inducers were used at a concentration of 2 mM: cellobiose (CB), cellotriose (CT), gentiobiose (GB), D-xylose (XL), and xylobiose (XB). No inductor was added to the control sample. Aliquots of mycelium suspension were taken after 1, 2, and 4 h of incubation.

RNA samples obtained from the mycelium were used for the reverse transcription reaction and quantified by qPCR (see Section 3.7 and Section 3.8) in order to determine the levels of transcription of *cbh1* (cellobiohydrolase 1), *egl2* (1,4-β-endoglucanase), and *bgl1* (β-glucosidase) genes.

Since the qPCR experiment was carried out in 96-well plates, a control sample was added to each plate to compare the data; genomic DNA of the B1-221-6 strain was used as a template. This also made it possible to compare the transcription levels of cellulases between themselves.

### 2.5. Effect of the Knockout of TF Genes on the Transcription of the cbh1, bgl1, and egl2 Genes

**Figure 3:**
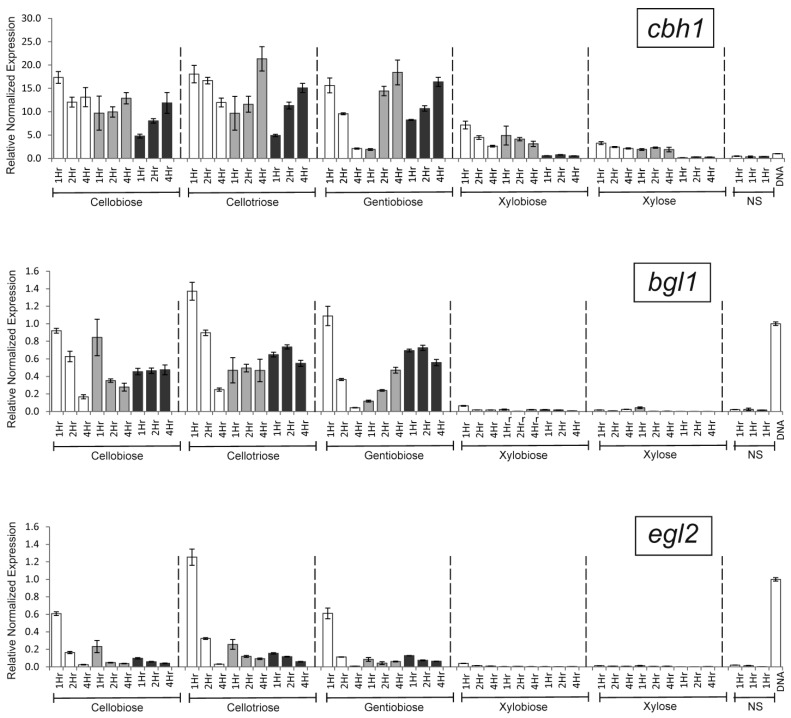
Relative normalized expression of the *cbh1*, *bgl1*, and *egl2* genes in the presence of various inducers for the B1-221-6 strain (white), B1-ΔclrB strain with a disrupted *clrB* gene (light grey), and B1-ΔxlnR strain with a knocked-out *xlnR* gene (dark grey). Induction time was 1, 2, and 4 h. Transcription levels of *cbh1*, *bgl1*, and *egl2* after 1 h of incubation without inducers (NS, No Sugar) are also shown. The far-right bar (‘DNA’) represents the control sample (43 ng of genome DNA from B1-221-6 as a template for qPCR), which was used to compare transcription levels between different plates.

The transcriptional analysis of the *cbh1* gene in the B1-221-6 strain showed that the selected inducers provided various degrees of transcription activation (Figure 3). The level of transcription in the presence of CB, CT, and GB was the same, while in the presence of XL and XB, it demonstrated a 4–5- and 2–3-fold reduction, respectively.

*clrB* gene knockout led to a significant change in the profile of *cbh1* gene transcription in the presence of GB. After one hour of cultivation with GB, a 7–10-fold reduction in transcriptional levels was observed. In the course of induction, the level of transcription in the wild strain dropped, while in the *clrB* knockout strain, it increased. At the fourth hour, *cbh1* transcription in the *clrB* knockout strain exceeded that in the wild-type strain by 7–10 times. The transcriptional profile also changed over time in the presence of CB and CT.

In the presence of XL and XB, knockout of the *xlnR* gene resulted in a strong decrease of *cbh1* transcription, equivalent to its basal level. In the case of CB, CT, and GB, a change in the transcriptional profile over time was observed. Thus, the level of transcription in the *xlnR* knockout strain after 1 h of induction in the presence of CB was 2–3-fold lower than in the B1-221-6 strain; however, in contrast to the B1-221-6 strain, it increased with time.

Unlike in the case of the *cbh1* gene, XL and XB induced a weak transcription of the *bgl1* gene (Figure 3). In the presence of other inducers (CB, CT, and GB), the corresponding knockouts of selected TF genes resulted in a slight (1–2.5-fold) decrease in the *bgl1* transcription level; the only exception was a *clrB* knockout strain incubated in the presence of GB. As in the case of the *cbh1* gene, after 1 h of cultivation with GB, the transcription drop was approximately 7–11-fold. In the course of further incubation, the level of transcription in the wild strain decreased, while in the *clrB* knockout strain, it increased. After 4 h, *cbh1* transcription in the *clrB* knockout strain was already 9–13 times higher than that in the wild strain.

Similar to the *bgl1* gene, XL and XB induced weak transcription of the *egl2* gene. In the presence of inducers such as CB, CT, and GB, disruption of each TF gene caused a decrease in the transcription level of the *egl2* gene (Figure 3). Thus, for the *clrB* and *xlnR* knockout variants, the decrease in transcription level that occurred after 1 h of incubation in the presence of CB was 3.5–4.4-, 2–3-, and 5–7-fold, respectively. A similar pattern was observed in the presence of CT. For GB, the maximum drop in the transcription level occurred when the *clrB* gene was knocked out.

### 2.6. Effect of the Overexpression of TF Genes on the Transcription of the cbh1, bgl1, and egl2 Genes

**Figure 4:**
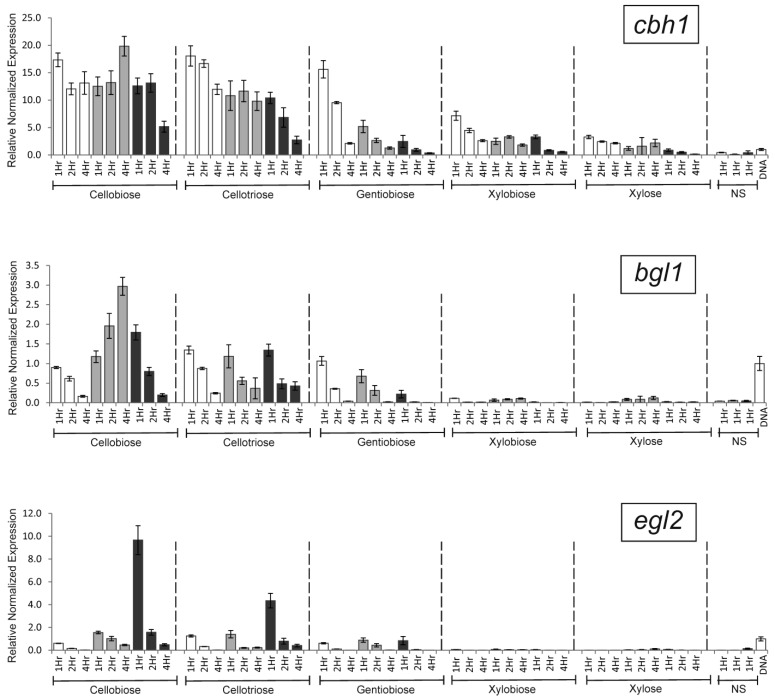
Relative normalized expression of *cbh1*, *bgl1*, and *egl2* genes in the presence of various inducers shown for the B1-221-6 strain (white), B1-ClrB strain with an overexpressed *clrB* gene (light grey), and B1-XlnR strain with an overexpressed *xlnR* gene (dark grey). Induction time was 1, 2, and 4 hrs. Transcription levels of *cbh1*, *bgl1*, and *egl2* genes determined after 1 h of incubation in the absence of inducers (NS, No Sugar) are also shown. The far-right bar (‘DNA’) represents a control sample (43 ng of B1-221-6 genome DNA as a template for qPCR), which was used to compare transcription levels between different plates.

Figure 4 shows the level of transcription of *cbh1*, *bgl1*, and *egl2* genes in recombinant strains with overexpressed *clrB* and *xlnR* genes in the presence of the same inducers.

Overexpression of *clrB* altered the *cbh1* transcription profile over time (Figure 4). In the other cases, overexpression of TF genes did not affect the transcription of the *cbh1* gene or had a negative effect. The maximum negative effect on *cbh1* gene transcription was observed for overexpression of *xlnR* in the presence of GB (after the first hour of induction, a 4–13-fold decrease was observed).

Overexpression of *clrB* significantly changed the *bgl1* transcriptional profile upon CB induction. After 1 h of induction, only a 1.1–1.5-fold increase in transcription was observed; however, after 4 h, it was already 14–23-fold.

Overexpression of *xlnR* also increased *bgl1* transcription in the presence of CB (Figure 4). During the first hour of induction, a 1.7–2.3-fold increase was observed. As in the case of the *cbh1* gene, a 3- to 10-fold decrease in transcriptional levels was observed upon GB induction.

Overexpression of all TFs caused an increase in transcription of the *egl2* gene upon induction by CB, CT, and XL (Figure 4). The maximum effect was observed in the presence of CB. After 1 h of induction, 2.3–2.9- and 13–19-fold increases were observed in the case of overexpression of *clrB* and *xlnR* transcriptional activators, respectively. Note that the effect of overexpression of *xlnR* in the presence of GB was less pronounced compared to that provided by CB and CT.

### 2.7. Transcriptome Analysis of Recombinant Strains

**Figure 5:**
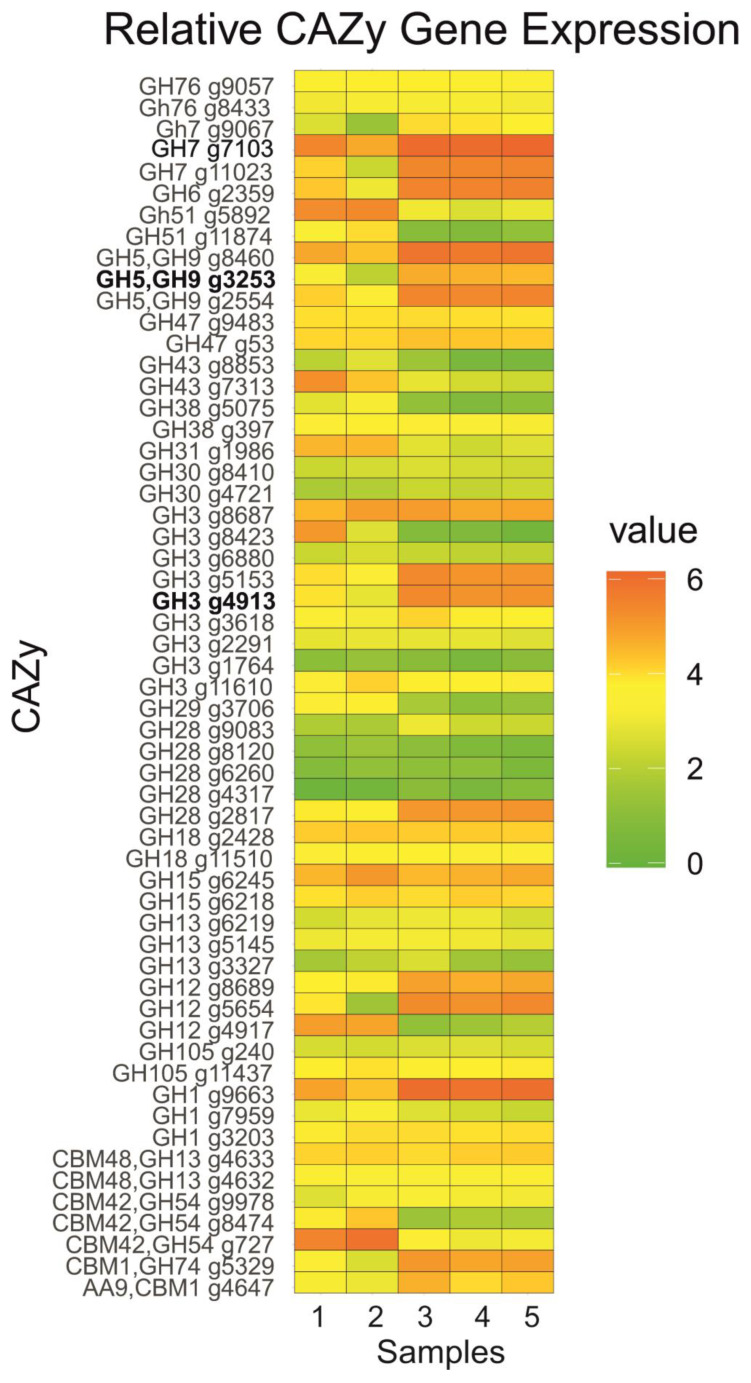
Heatmap of CAZy enzyme gene expression profiles across five transcriptomic samples (2 h after induction). The data were log-transformed to normalize expression values and reduce the impact of outliers. Colors represent the relative expression levels, with green indicating no detectable expression and red representing the highest expression levels observed. Samples: 1, B1-221-6 in the presence of xylose; 2, B1-ΔxlnR in the presence of xylose; 3, B1-221-6 in the presence of cellobiose; 4, B1-ΔclrB in the presence of cellobiose; 5, B1-ΔxlnR in the presence of cellobiose. Sequences encoding transcripts of *cbh1* (g7103), *bgl1* (g4913), and *egl2* (g3253) are indicated in bold.

The results of the transcriptome analysis for the B1-221-6 and B1-ΔxlnR strains induced by xylose and cellobiose, as well as B1-ΔclrB strains induced by cellobiose, shown for glycoside hydrolases of *P. verruculosum*, are presented in Figure 5. The choice of inducers was determined by the maximum effects of changes in relative normalized expression determined by qPCR. RNA taken at the second hour after induction was used as the material for such analyses.

According to this figure, the majority of glycoside hydrolases (GHs) with high transcriptional levels were more significantly induced by cellobiose than xylose. For this group of GHs, the knockout of the *xlnR* gene caused a decrease in the transcriptional level upon induction by xylose, which indicates the effect of XlnR during growth on xylose. Upon induction by cellobiose, the knockout of the *xlnR* gene did not affect or had a weak effect on the transcriptional level. Note that the *cbh1* (g7103), *bgl1* (g4913), and *egl2* (g3253) genes analyzed by qPCR belong to this group.

Other GHs with high transcriptional levels include α-L-arabinofuranosidases (g727 and g5892) and endo-α-L-arabinase (g7313), which are induced to a greater extent by xylose. The knockout of *xlnR* under xylose induction had different effects: it increased transcription for g727, decreased it for g7313, and did not affect g5892.

### 2.8. Analysis of Specific Activities of Dried Enzyme Preparations Obtained After 144 h of Cultivation

The data obtained after 144 h of fermentation of recombinant strains with knockout and overexpression of TFs under the standard cultivation scheme followed by drying of enzyme preparations represent the greatest interest in the practical aspect. The fermentation scheme was developed earlier for *P. verruculosum* strains [26] and represents a fed-batch scheme with three additions of microcrystalline cellulose after 48, 72, and 96 h of fermentation. The resulting enzymatic activities for the dried forms of enzyme preparations (EPs) are presented in Table 1.

According to Table 1, a 4-fold decrease in EP activity was observed in the B1-ΔclrB strain for CMC and MCC, while insignificant decreases were revealed for pNPG and xylan. At the same time, for the constitutive expression of ClrB, this activity doubled for pNPG and also increased for CMC and xylan, although it did not recover to the values of the control B1-221-6 strain. For the B1-ΔxlnR strain, all enzymatic activities decreased to varying extents; however, in the case of the constitutive expression of *xlnR*, they were restored to the level of the original B1-221-6 strain (for CMC and MCC) or demonstrated a 2- and 3.5-fold increase for pNPG and xylan, respectively.

## 3. Materials and Methods

### 3.1. Strains and Media

*Penicillium verruculosum* B1-221-6 (VKM F-382, All-Russian Collection of Microorganisms, Puschino, Russia) was used as a host for knocking out the TF genes as it was previously described for knocking out the TF TacA [26]. *P. verruculosum* B1-537 (VKM F-3972D, All-Russian Collection of Microorganisms, Puschino, Russia) was used as an auxotrophic host strain (Δ*niaD*) for transformation to produce clones overexpressing the TF genes as it was described for a series of genes previously [27]. The original *P. verruculosum* strain is a xylotrophic fungus, secreting a complex of cellulolytic enzymes. This fungus was collected from a stub in the forests of western Siberia in 1995.

*Escherichia coli* MachI T1^R^ (Thermo Fisher Scientific Inc., Waltman, MA, USA) was used in the subcloning experiments.

The minimal medium (MM) contained (g/L): (NH_4_)_2_SO_4_—5.0; KH_2_PO_4_—15.0; MgSO_4_—0.6; CaCl_2_—0.6; FeSO_4_⋅7H_2_O—0.005; MnSO_4_⋅H_2_O—0.0016; ZnSO_4_⋅7H_2_O—0.0014; CoCl_2_—0.002; and glucose—10.0. The medium was supplemented with different nitrogen sources (10 mM NaNO_3_ for *niaD^+^* or 10 mM NH_4_Cl for Δ*niaD* strains). In addition, 2% of Bacto agar was added for plate cultivation.

The complete medium (CM) was the same as the MM, with the addition of yeast extract (2 g/L) and Bacto Peptone (3 g/L).

The fermentation medium (FM) contained (g/L): KH_2_PO_4_—10; K_2_HPO_4_—1; (NH_4_)_2_SO_4_—5; CaCl_2_×2H_2_O—0.3; MgSO_4_×7H_2_O—0.3; cellulose—40; yeast extract—10; and wheat bran—10.

### 3.2. Genome Sequencing and Assembly

A sample of genomic DNA from *P. verruculosum* strain B1-221-6 was prepared for sequencing using the Illumina DNA Prep Kit (Illumina Inc., San Diego, CA, USA). The quality of the resulting libraries was evaluated with the Fragment Analyzer (Advanced Analytical Technologies Inc., Ames, IA, USA). After quality control and DNA quantification, the library was sequenced on an Illumina MiSeq v2 platform (read length: 250 bp, paired-end; Illumina Inc., San Diego, CA, USA). FASTQ files were generated using the bcl2fastq v2.20 Conversion Software (Illumina Inc., San Diego, CA, USA), yielding 4,948,540 paired reads. The quality of the sequencing reads was analyzed using a FastQC program [28].

Genome assembly was performed using the SPAdes assembler (version 3.15.4) with the error correction module enabled [29]. The final assembly resulted in a genome size of 32.968 Mb, represented by 281 contigs. The maximum contig length was 1,926,302 base pairs.

### 3.3. Knockout of TF Genes by CRISPR/Cas9

In the pSgRNAniaD plasmid [22], which carries a Cas9 guide RNA (sgRNA) with a spacer for the *niaD* gene encoding *P. verruculosum* nitrate reductase under the control of the *A. niger* 5S RNA promoter, the spacer for the *niaD* gene was replaced by the specific spacers for the *clrB* and *xlnR* genes; this operation was performed using PCR mutagenesis with the primers specified in Table S1 and pSgRNAniaD as a template. The PCR fragments were treated with *Dpn*I endonuclease (Thermo Fisher Scientific Inc., Waltman, MA, USA) and used for the transformation of *E. coli* MachI cells. Isolated plasmid DNA was sequenced with a m13/pUC reverse primer (Thermo Fisher Scientific Inc., Waltman, MA, USA).

The *Bam*HI–*Sal*I fragment of plasmids carrying Cas9 sgRNA with a spacer for TF genes under the control of 5S RNA *A. niger* was inserted into the pGpdCas9 plasmid [22] containing the *cas9* gene. As a result, pGpdCas9sgXlnR and pGpdCas9sgClrB plasmids were obtained to knock out the *xlnR* and *clrB* genes, respectively.

Knockouts of TF genes were carried out by co-transforming *P. verruculosum* B1-221-6 with the resulting plasmids, together with the pSgRNAniaD plasmid, as described earlier [22,26].

The transformants were selected on the MM with the addition of 10 mM NH_4_Cl as a nitrogen source and 0.6 M NaClO_3_ as a selective substance. Positive colonies were transferred on MM plates with 10 mM NH_4_Cl or 10 mM NaNO_3_ as a nitrogen source but without NaClO_3_. Clones able to grow on NaNO_3_ were rejected from further analysis [22].

Then, genomic DNA was isolated from clones chosen on a selective medium. Isolated DNA was used to amplify sequence fragments containing the protospacer region (Table S1).

### 3.4. Constitutive Expression of TF Genes

The coding regions of cloned TFs were fused to the homologous constitutive *gpdA* promoter and *cbh1* terminator using a Gibson Assembly^®^ Master Mix (New England Biolabs, Ipswich, MA, USA) by replacing the *cas9* gene in the pGpdCas9 plasmid [22].

Using the online NEBuilder Assembly tool v2.5.0 (https://nebuilder.neb.com/#!/, accessed on 7 December 2024), fusing primers were selected (Table S1). The corresponding regions were amplified using a high-precision Phusion Hot Start II DNA Polymerase (Thermo Fisher Scientific Inc., Waltman, MA, USA).

PCR fragments were treated with 10 U of DpnI endonuclease (Thermo Fisher Scientific Inc., Waltman, MA, USA) for 1 h and then purified from the reaction mixture by a PCR purification kit (QIAGEN, Valencia, CA, USA).

A mixture of PCR fragments of the vector and amplified TF genes was treated with a Gibson Assembly Master Mix (New England Biolabs, Ipswich, MA, USA), and then transformed into *E. coli* MachI T1^R^ competent cells. Plasmid DNA was isolated from grown colonies and used as a template for PCR screening with the corresponding primers (Table S1). Amplified TF genes were sequenced to check for correct assembly and the absence of mutations in the coding sequence (CDS).

Protoplasts of the host *P. verruculosum* B1-537 (ΔniaD) strain were co-transformed by the resulting plasmids, together with a selective plasmid, pSTA-10 [25], using the modified method described by Aleksenko et al. [24]. The pSTA-10 plasmid contained a nitrate reductase gene, providing complementation for a defective *niaD* gene in the host strain. This enables the selection of transformants on minimal media supplemented with 10 mM NaNO_3_.

Genomic DNA was isolated from the mycelium of selected clones and was used to determine the relative number of integrated copies of the target plasmid using the qPCR method. Forward primers bound to the *gpdA* gene promoter, and reverse primers bound to the coding part of the corresponding TF (Table S1).

### 3.5. Induction of cbh1, egl2, and bgl1 Gene Transcription

Induction of the transcription of the cellulase genes, encoding CBHI, EGL2, and BGL1, was measured as described previously [19]. Briefly, spore suspensions of the *P. verruculosum* strains (~3 × 10^6^ spores) were collected into a 750 mL Erlenmeyer flask containing 100 mL of MM with 0.8% glucose, 50 mM citrate-phosphate (Ci-PO_4_) buffer (pH 5.6), and 10 mM NH_4_Cl as a nitrogen source, and incubated on an orbital shaker (220 rpm) at 32 °C for 48 h. Then, the mycelium was separated from the culture liquid on a filtration glass filter (Millipore, Burlington, MA, USA) to remove glucose and washed with MM containing 50 mM Ci-PO_4_ buffer and 10 mM NaNO_3_. After this, the mycelium was resuspended and transferred into 10 glass tubes in equal volumes. Then, the test tubes containing the mycelium suspension were incubated on an orbital shaker under the same conditions to utilize the glucose residues and turn off the carbon catabolite repression (CCR) mechanism. After 60 min of incubation, 1 mL of a 10 mM induction sugar solution was added. No sugar was added to the control tube. The tubes were incubated on an orbital shaker under the same conditions, with the selection of 1.2 mL samples of the mycelial suspension from each tube after 1, 2, and 4 h of incubation.

### 3.6. RNA and DNA Isolation

The mycelial suspension (1.2 mL) was transferred into 2 mL tubes containing 500 μg of glass beads with a diameter of 0.5 mm (QIAGEN, Valencia, CA, USA). The tubes were centrifuged for 10 min at 5000× *g*, and the supernatant was removed. TRIzol™ Reagent (Thermo Fisher Scientific Inc., Waltman, MA, USA) was used for RNA isolation [30]. A FastPrep-24 homogenizer (MP Biomedicals LLC, Irvine, CA, USA) was used for mycelium disruption. RNA concentration was measured using a NanoDrop (Thermo Fisher Scientific Inc., Waltman, MA, USA). RNA quality was determined by electrophoresis in 1% agarose gel.

DNA was isolated using “DNeasy Plant Kit” (QIAGEN, Valencia, CA, USA) according to the manufacturer’s recommendations.

### 3.7. Reverse Transcription and Real-Time Polymerase Chain Reaction (qPCR) Assays

Total RNA samples (1 μg) were treated with “RNAse-free DNAse I” (Thermo Fisher Scientific Inc., Waltman, MA, USA) according to the manufacturer’s recommendations. The cDNA was synthesized on treated RNA (3 μL) using a “RevertAid H Minus First Strand cDNA Synthesis Kit” (Thermo Fisher Scientific Inc., Waltman, MA, USA), as well as a “Random hexamer primer” and an “Oligo(dT)18 primer” (Thermo Fisher Scientific Inc., Waltman, MA, USA). After completion of the reverse transcription reaction, the samples were diluted with 150 μL of DEPT water. The resulting cDNA was used as a template for qPCR. *P. verruculosum* actin (*actA*) and glyceraldehyde-3-phosphate dehydrogenase (*gpdA*) genes were used as reference genes for qPCR. We used two reference genes for internal validation to increase the reliability of the obtained results.

The qPCR reaction mixture consisted of 0.5 pM of forward and reverse primers for *bgl1* and *egl2 P. verruculosum* (Table S1), as well as 8 μL of a «2.5× reaction mix for qPCR with EVA Green I dye» (Syntol LLC, Moscow, Russia), 10 μL of the analyzed cDNA sample, and water (up to 20 μL of the total sample volume).

The qPCR reaction mixture for *actA*, *gpdA*, and *cbh1 P. verruculosum* contained 0.4 pM of forward primers, 0.4 pM of reverse primers, and 0.4 pM of a TaqMan probe for each gene (Table S1), as well as 8 μL of a «2.5× reaction mix for qPCR» (Syntol LLC, Moscow, Russia), 10 μL of the analyzed cDNA sample, and water (up to 20 μL of the total sample volume).

To compare the Cq values (the number of cycles required to exceed the background fluorescence level) of different plates, identical samples of the genomic DNA of the 221-6 strain were loaded on each plate and for each gene in triplicate.

qPCR was performed under the following conditions: first stage, 5 min at 95 °C; second stage, 39 cycles, 15 s at 95 °C and 45 s at 60 °C, with fluorescence measurements conducted after each cycle; the third stage included a temperature increase from 75 °C to 95 °C at a rate of 0.2 °C/10 s steps with the fluorescence level measurement. The melting curve of the PCR products was plotted in the third stage.

Amplification was carried out in a CFX96 amplifier (Bio-Rad Laboratories Inc., Hercules, CA, USA) in compatible 96-well low-profile plates. The results were analyzed using Bio-Rad CFX Manager v.3.1 software.

All samples were analyzed in triplicate.

### 3.8. Transcriptome Sequencing and Differential Expression Analysis

RNA sequencing services were provided by Genomed ltd (Moscow, Russia). The cDNA library was sequenced on an Illumina MiSeq sequencing system (Illumina, Inc., San Diego, CA, USA). Reads obtained from the sequencing machine included raw reads containing adapters or low-quality bases. High-quality clean reads were obtained by eliminating reads with adapters, reads with more than 10% unknown nucleotides, and reads with low-quality bases with Q values ≤ 20. The quality check was performed on fastq-formatted raw data using SOAPnuke 2.1.5 software for quality filtering and trimming [31]. The cleaned raw reads were used for further analysis.

The clean data obtained from sequencing were mapped on the reference genome of *P. verruculosum* using HISAT2 2.1.0 [32]. The generated SAM files were converted to BAM files using the SAMtools package [33]. Data analysis was performed using FeatureCounts 2.0.5 software [34] to quantify gene expression levels.

Differential expression analysis of conditions was performed using the DEGSeq R package (1.12.0) [35]. Relative expression was measured in the form of CPM (counts per million) to normalize it for the library size and to enable comparisons across samples.

Normalized expression values were transformed to the log2 scale to reduce the influence of outliers and to emphasize relative differences in expression levels [36]. To visualize the expression profiles of CAZy-annotated genes across the samples, a heatmap was constructed using the ggplot2 package (v3.4.0) [37] in R. Normalized expression values (CPM) were log-transformed to reduce the influence of outliers and emphasize relative differences.

### 3.9. Enzymatic Activity Assays and Protein Concentration Measurement

The samples of culture fluids were taken out after 144 h of cultivation in 1 L KF-108/3 fermenters (Prointech, Puschino, Russia), as described earlier [38]. The cultural fluids were screened via measurement of enzymatic activity.

Enzymatic activities against polymeric substrates, such as beech xylan (Megazyme, Wicklow, Ireland), were evaluated via the determination of the release of reducing sugars using the Nelson–Somogyi method [39] with some modifications [40]. For the determination of endoglucanase and avicelase activities, carboxymethyl cellulose (CMC) (Sigma-Aldrich Inc., St. Louis, MO, USA) and Avicel PH-105 cellulose (MCC) (Serva, Heidelberg, Germany) were used as substrates, respectively.

β-Glucosidase activity was determined by the hydrolysis of p-nitrophenyl-β-D-glucopyranosid (pNPG) (Sigma-Aldrich Inc., St. Louis, MO, USA), as described by Osipov et al. [38].

### 3.10. Statistical Analysis

All experiments and measurements were performed in triplicate. Significant differences were considered if *p*-values were below 0.05. Measurement errors in qPCR were calculated using Bio-Rad CFX Manager v.3.1 software (Bio-Rad Laboratories Inc., Hercules, CA, USA). Quantitative results of the enzyme activity measurements were analyzed using STATISTICA v. 6.1 software (StatSoft Inc., Tulsa, OK, USA).

## 4. Discussion

Transcription factors of the Zn(II)2Cys6 class, such as CLR-1/ClrA, CLR-2/ClrB/ManR, and XlnR/Xyr1, are known to regulate the transcription of cellulases in *T. reesei*, *N. crassa*, *A. nidulans*, *Penicillium oxalicum* [41], and some other fungi.

We cloned the closest homologues of ClrB and XlnR TFs from *P. verruculosum*. Note that we were unable to identify a CLR-1/ClrA homologue. The goal of our study was to determine the role of each TF in the transcription of the main cellulase genes (*cbh1*, *egl2*, and *bgl1*) encoding key secreted cellulases (CBH1, EGL2, and BGL) in the filamentous fungus *P. verruculosum.*

This study was carried out by knocking out the genes of the corresponding TFs via introducing frameshift mutations in the 5′-region of the genes (upstream of the Zn-finger domain) using CRISPR/Cas9 technology, and analyzing their constitutive expression under the control of the constitutive promoter of the *gpdA* gene encoding glyceraldehyde 3-phosphate dehydrogenase from *P. verruculosum*. One strain from each knockout and overexpressed TF gene, as well as the control strain B1-221-6, was used to assess the transcriptional regulation of cellulase genes.

Transcriptional analysis by qPCR showed that the *cbh1*, *bgl1*, and *egl2* genes responded differently in the corresponding TF knockout/overexpression strains to different inducers but shared common gene regulation patterns.

Amore (2013) showed that the pattern of induction, i.e., the specificity of inducers towards cellulase genes, differs between *T. reesei*, *N. crassa*, and *Aspergillus* spp. [42]. In this study, we demonstrated that the induction of cellulases in *P. verruculosum* follows yet another species-specific pattern. In the B1-221-6 strain, *cbh1*, *egl2*, and *bgl1* are induced by CB, CT, and GB, but XL and XB significantly induced only the *cbh1* gene.

The knockout of the *xlnR* gene in the presence of XL and XB led to an almost complete cessation of *cbh1* transcription throughout the experiment. This suggests that XlnR is a key TF for *cbh1* transcription in the presence of these inducers. XL and XB showed a similar pattern of regulation, with a 2-fold difference in transcription due to the fact that XL was a monosaccharide, while XB was a disaccharide, and they were added in equimolar concentrations. In the case of CB, CT, and GB, changes in the transcription profile of the *cbh1* and *bgl1* genes were observed over time, which implies the role of XlnR in the transcription of these genes in the presence of these inducers. Decreased transcription of the egl2 gene is a consequence of the influence of XlnR.

Overexpression of *xlnR* in the presence of CB led to a slight (1.7–2.3-fold) increase in the transcription of the *bgl1* gene and a significant (13–19-fold) increase in the transcription of the *egl2* gene. In addition, a significant (3–5-fold) increase in transcription was observed for the *egl2* gene in the presence of CT.

The knockout of the *clrB* gene in the presence of CB and CT resulted in a decrease in the transcription of only the *egl2* gene, while *clrB* overexpression caused a slight increase in its transcription. Interestingly, *clrB* overexpression altered the transcriptional profile of the *bgl1* gene in the presence of CB. After 4 h of induction, we observed a 14–23-fold increase in transcription.

To confirm the data obtained by qPCR, transcriptomes of the B1-221-6, B1-ΔxlnR, and B1-ΔclrB strains were obtained 2 h after induction with cellobiose and xylose. Analysis of transcription data for the *cbh1*, *egl2*, and *bgl1* genes showed the reliability of qPCR data (Figure 5). *cbh1* was strongly induced by xylose in contrast to the weak transcription of *bgl1* and *egl2*. We assume that XlnR strongly binds to the promoter of the *cbh1* gene, while XlnR’s influence is rather insignificant in the case of the xylose induction of the *bgl1* and *egl2* genes. To search for the recognition sites in the promoter regions of the *cbh1*, *bgl1*, and *egl2* genes, we used Vector NTI v10.1.1 software (Thermo Fisher Scientific Inc., Waltman, MA, USA). Analysis of 2 kb promoter regions of the *cbh1*, *bgl1*, and *egl2* genes showed the presence of eight, two, and one consensus regions for the XlnR binding site 5′-GGCWAW-3′ [43], respectively.

The heatmap shows the effect of an *xlnR* knockout on *cbh1* transcription in the presence of xylose. Transcriptome results also showed that *egl2* and *bgl1* genes responded to the *xlnR* knockout. Due to the low transcription levels, this fact was difficult to highlight in the qPCR diagrams.

The effect of TF knockouts on the *egl2* gene in the presence of CB is poorly visible in the heatmap, though digital transcriptome data (Table S2) show a 1.7- to 1.8-fold decrease in *egl2* transcription in knockout strains.

Thus, we confirmed that XlnR regulates the transcription of cellulase genes in *P. verruculosum*, as it does in *A. niger* and *P. oxalicum* and as its homologue Xyr1 does in *T. reesei* [12]. In the case of major cellulase genes, XlnR was the main TF in the presence of XL and XB.

In *N. crassa*, *A. nidulans*, and *A. oryzae*, Clr-2/ClrB acts as the major regulator of cellulolytic enzyme genes [44]. In *P. verruculosum* (anamorph *Talaromyces verruculosus*), the role of ClrB appeared to be rather minor, as in the case of the Clr2-like protein in *Talaromyces cellulolyticus*, which represents a ClrB homologue. In this fungus, deletion of the *tclB2* gene encoding this protein did not affect cellulase activity when grown on Avicel [45]. The existence of different regulatory modes leading to the efficient regulation of cellulase/hemicellulase production in filamentous fungi was also described in a comparison of *T. reesei* and *P. oxalicum* cellulose-degrading strains [46].

The practical output of this study is an increase in the activity of dried EPs (i.e., ready-to-use samples for the bioconversion of plant raw materials, etc.) toward xylan and pNPG in the B1-XlnR strain cultivated under standard fermentation conditions for *P. verruculosum* strains [4]. Constitutive expression of the transcription factor XlnR in the B1-XlnR strain leads to an increase in xylanase and β-glucosidase activity by 3.5- and 2-fold, respectively, relative to the corresponding activities in the B1-221-6 strain. It is interesting that this effect was not observed for *T. cellulolyticus*, in which XlnR was also overexpressed [47]. In our case, the presence of xylan-rich wheat bran in the fermentation medium may provide a source of the inducer for XlnR, which promotes the biosynthesis of xylanase and β-glucosidase.

## 5. Conclusions

The studied TFs (ClrB and XlnR) affect the transcription of key cellulase genes in *P. verruculosum*, but with varying specificities. Both TFs had a significant effect on the *egl2* gene, as demonstrated in experiments with the knockout and constitutive expression of TF genes. As for the *bgl1* gene, their influence was less significant, and in the case of the *cbh1* gene, the effect was rather insignificant with the exception of TF XlnR in the presence of D-xylose and xylobiose.

Note that the performed study has a significant biotechnological potential related to the use of a filamentous fungus, *P. verruculosum*, as a host for the production of industrially important enzymatic preparations [4]. This fungal expression system is based on the use of regulatory elements of the *cbh1* gene, so the determination of factors influencing its transcription represents an important task. Our recent genome data for *P. verruculosum*, as well as the obtained data on its transcriptomes in the presence of various disaccharide inductors, provide us with the opportunity to clarify the key TFs, especially for the transcription and expression of the *cbh1* gene.

## Figures and Tables

**Table 1:** Specific activities (U/g of protein) of enzyme preparations (EPs) produced by recombinant strains with knocked out and overexpressed TFs.

EP	MCC ^1^	CMC ^2^	pNPG ^1^	Xylan ^2^
B1-221-6	390 ± 22	10,280 ± 500	1700 ± 100	21,400 ± 800
B1-ΔclrB	100 ± 11	2400 ± 80	1200 ± 60	19,400 ± 90
B1-ΔxlnR	150 ± 9	2100 ± 60	1100 ± 50	500 ± 70
B1-ClrB	360 ± 9	4900 ± 60	3500 ± 50	12,600 ± 90
B1-XlnR	330 ± 5	11,000 ± 400	3400 ± 60	79,100 ± 80

^1^ 40 °C, pH 5.0.; ^2^ 50 °C, pH 5.0.

## Data Availability

The authors declare that the data supporting the findings of this study are available within the main text of the manuscript. Raw data available from the corresponding authors upon reasonable request.

## Supplementary Materials

The following supporting information can be downloaded at: https://www.mdpi.com/article/10.3390/ijms252413373/s1.
