# Supplementary material for: Transcription Factors ClrB and XlnR and Their Effect on the Transcription of Cellulase Genes in the Filamentous Fungus Penicillium verruculosum
Source: Int J Mol Sci. 2024 Dec 13;25(24):13373. doi: 10.3390/ijms252413373 (PMC11678864; doi:10.3390/ijms252413373)
Supplement: Supplementary file 1 [file ijms-25-13373-s001.zip › ijms-3363768-supplementary.pdf]

SUPPLEMENTARY MATERIALS

Table S1. Primers used in this study

Gene	Primer name	5`-3`	Use
clrB	CLRBSR2 [2]	GCGGAGACATGTGTTTCTACAGACG	
	clrB_65sgRF	CTACAACAAGACGAAGCGAGGTTTTAGAGCTAGAAATAGCAAG	Change spacer for knockout
	clrB_65sgRR	CTCGCTTCGTCTTGTTGTAGCTATTCGTCCTTTCATACAACAG	
	CLRBGF [1]	CAACTATCACCGCAATCATGTTTCTCACATTCGAGTCC	Amplification for GibsonAssembly
	CLRBGR	GGAGGGCGACACAGTCTCACTGGATCCTAGCATCG	
xlnR	XLNRSR2 [1, 2]	GCCTCGGATTCTGATGCCATC	
	xlnR219sgRF	GCATTCTGCTGATACGGTTGGTTTTAGAGCTAGAAATAGCAAG	Change spacer for knockout
	xlnR219sgRR	CAACCGTATCAGCAGAATGCCTATTCGTCCTTTCATACAACAG	
	XLNRGF	CAACTATCACCGCAATCATGGCACAACCGTCGCAAAC	Amplification for GibsonAssembly
	XLNRGR	GGAGGGCGACACAGTCTCACAAAGCTAATCCGCTGC	
vector	CBGPVGF	GACTGTGTCGCCCTCCTC	Amplification for GibsonAssembly
	CBGPVGR	GATTGCGGTGATAGTTGCTTAG	
gpdA	PVGPDQF2	AACGGCAAGCGCGTCAAGTTCT	qPCR
	PVGPDQR2	ACCCTTCAAGTGAGCAGAGGCCTT	
	PVGPDRX	ROX-CCGCCAACATCAAGTGGTCCGAGAC-BHQ2	TaqMan probe
	prGpdSF[2]	GCTGCGTTACTTCTGGTGGAG	
actA	PVACTQF1	ACAAGAAATCCAGACCGCTTCCC	qPCR
	PVACTQR1	TTTCGAGACCGATGACGGAAGG	
	PVACTFM	FAM-CCCGACGGACAGGTCATCACCATTG-BHQ1	TaqMan probe
cbh1	PVCBHQF2	CAACATGGGCGCTGCTCTCAAA	qPCR
	PVCBHQR2	GAAGTTGTAGGGCAGGAACCACGA	
	PVCBHC5	CY5-CGACAGCACTTACCCCACAAACGCAA-BHQ2	TaqMan probe
bgl1	PVBGLQF2	TAGCGTTACCACTGGCCCAACTACTG	qPCR
	PVBGLQR2	TGAGCAACCTCCGAACCTGTAACG	
egl2	PVEG2QF1	CGCCTGGACTTGGACAACTTACAAC	qPCR
	PVEG2QR1	GCTTACGCATTGGTCTGATGTGC	

[1] Primers were used for amplification and sequencing protospacer region
[2] Primers were used for detection relative plasmid copy number

Table S2. Normalized expression levels of key cellulase genes (*cbh1*, *egl2*, and *bgl1*) across five transcriptomic samples, measured in CPM (Counts Per Million). The table shows normalized expression levels for *cbh1*, *egl2*, and *bgl1* genes. Higher CPM values indicate stronger gene expression, with distinct variations observed between experimental conditions

**Gene**	**Transcript ID**	**Strain**				
		**B1-226-1 (XL)**	**B1-ΔxlnR (XL)**	**B1-226-1 (CB)**	**B1-ΔclrB (CB)**	**B1-ΔxlnR (CB)**
cbh1	g7103	319 667	69 895	1 444 811	1 758 318	1 647 659
bgl1	g4913	10 030	923	280 901	163 823	193 491
egl2	g3253	2 463	160	60 978	35 734	36 288

```
# TBLASTN 2.12.0+
# Query: XP_663412.1 protein clrA [Aspergillus nidulans FGSC A4]
# Database: Genome
# 0 hits found
# BLAST processed 1 queries

# TBLASTN 2.12.0+
# Query: XP_660973.1 protein clrB [Aspergillus nidulans FGSC A4]
# Database: Genome
# Fields: subject acc., s. start, s. end, subject length, evalue, bit score, % identity
# 3 hits found
NODE_19_length_558701_cov_33.686249,346313,347377,558701,2.39e-149,284,44.474
NODE_19_length_558701_cov_33.686249,347425,348276,558701,2.39e-149,216,40.070
NODE_19_length_558701_cov_33.686249,346009,346227,558701,2.39e-149,70.5,51.948
# BLAST processed 1 queries

# TBLASTN 2.12.0+
# Query: XP_680879.1 protein xlnR [Aspergillus nidulans FGSC A4]
# Database: Genome
# Fields: subject acc., s. start, s. end, subject length, evalue, bit score, % identity
# 5 hits found
NODE_4_length_953474_cov_33.829619,161230,158618,953474,0.0,882,60.586
NODE_1_length_1926322_cov_33.970848,1152424,1151036,1926322,5.45e-110,306,42.457
NODE_1_length_1926322_cov_33.970848,1153022,1152477,1926322,5.45e-110,111,45.161
NODE_20_length_552516_cov_33.303674,482615,483772,552516,1.80e-64,224,38.810
NODE_20_length_552516_cov_33.303674,483841,483990,552516,1.80e-64,42.7,47.059
# BLAST processed 1 queries

# TBLASTN 2.12.0+
# Query: XP_011394265.1 C6 finger domain-containing protein [Neurospora crassa OR74A]
# Database: Genome
```

```
# 0 hits found
# BLAST processed 1 queries

# TBLASTN 2.12.0+
# Query: XP_962712.2 fungal specific transcription factor domain-containing protein [Neurospora crassa OR74A]
# Database: Genome
# Fields: subject acc., s. start, s. end, subject length, evalue, bit score, % identity
# 2 hits found
NODE_19_length_558701_cov_33.686249,346262,347362,558701,7.86e-84,195,35.620
NODE_19_length_558701_cov_33.686249,347440,347877,558701,7.86e-84,135,46.939
# BLAST processed 1 queries

# TBLASTN 2.12.0+
# Query: XP_962611.1 transcriptional activator xlnR [Neurospora crassa OR74A]
# Database: Genome
# Fields: subject acc., s. start, s. end, subject length, evalue, bit score, % identity
# 2 hits found
NODE_4_length_953474_cov_33.829619,160276,158618,953474,0.0,602,60.377
NODE_1_length_1926322_cov_33.970848,1152124,1151036,1926322,3.59e-88,310,47.411
# BLAST processed 1 queries
```

Figure S1. Results of comparison of amino acid sequences of TFs and ClrA, ClrB, XlnR of *A. nidul*ans (strain FGSC A4, GeneBank ID: XP_663412.1, XP_660973.1, and XP_680879.1) and Clr-1, Clr-2, XlnR from *N. crassa* (strain OR74A, GeneBank ID: XP_011394265.1, XP_962712.2, and XP_962611.1,) with *P. verruculosum* genome data using the tblastn program from the BLAST+ package version 2.12.0.

A

```
1      GCTAGTCGACTCCGCATTTATGTGTCTAGACCTGGTCCCTGGGAGGGTATACAATGTATGTCACGACATACAAGCTCACTCGAGACCCATTCGGTGATT
                                   M  F  L  T  F  E  S  S  Q  P  S  N  K  N  G  L  R  N  P  K  Q  T  T  T  R ·
100    GTGTCTCATACTCTGGCTCAGCAGAATGTTTCTCACATTCGAGTCCTCCCAACCCAGCAACAAAAACGGCCTTCGCAACCCTAAACAAACTACAACAAG
       · R  S  E  R  L  N  R  R  R  R  S  T  P  R  A  C  T  S  C  R  Q  R  K  I  R  C  D  G  E  K  P  C  E ·
199    ACGAAGCGAGCGGCTCAACCGACGACGCCGCTCCACGCCGCGAGCATGTACTTCATGCCGTCAACGGAAAATTAGATGCGATGGCGAGAAGCCCTGTGA
       · A  C  R  W  Y  K  K  A  E  Q  C  S  Y  P  E  R  E  R  E  R  *  V  I  K  S  G  G  R  P  K  E  R  D ·
298    AGCTTGTCGGTGGTACAAGAAGGCTGAGCAATGTAGCTACCCGGAACGGGAGAGAGAGAGGTAAGTCATAAAATCGGGGGGACGACCAAAGGAAAGAGA
                            H  S  E  E  T  S  S  L  P  D  Y  R  A  A  L  E  R  L  F  P  E  T  A  P  E  N  I ·
397    TATGAACTCAACGATATAGTCACTCAGAAGAGACATCATCATTACCGGATTATCGAGCAGCGTTAGAACGGCTATTTCCCGAGACAGCGCCTGAGAATA
       ·  V  N  L  S  R  E  R  L  L  A  L  I  S  K  P  T  D  G  S  H  Y  S  A  Q  S  Q  H  Q  D  S  L  T  I ·
496    TCGTGAACCTATCGAGGGAGAGATTGCTAGCTTTGATATCAAAGCCTACGGATGGATCTCATTACTCTGCTCAGTCCCAACATCAAGATTCATTGACAA
       ·  A  T  S  A  S  V  E  T  H  V  S  A  L  S  M  E  R  P  G  L  E  S  L  H  A  I  P  G  E  Q  E  Q  L ·
595    TCGCCACATCCGCGTCTGTAGAAACACATGTCTCCGCATTGTCAATGGAAAGGCCAGGTCTGGAGTCTCTGCATGCGATACCAGGAGAACAAGAACAAC
       ·  D  E  T  Q  C  A  S  A  S  E  E  S  E  E  H  I  S  D  D  V  N  A  L  S  L  P  A  R  N  L  T  S  Y ·
694    TCGATGAAACCCAGTGTGCTAGCGCGTCTGAAGAGTCAGAAGAGCATATATCAGACGACGTCAATGCGTTGTCTCTACCAGCTCGGAATCTTACTTCAT
       ·  L  G  V  S  S  I  Q  A  A  L  K  V  I  A  W  L  H  P  E  L  N  A  H  L  S  S  P  K  D  Q  R  H  H ·
793    ATCTGGGCGTATCGTCTATTCAGGCTGCGCTCAAAGTCATTGCGTGGCTCCATCCGGAATTGAATGCACATCTTAGTTCTCCCAAAGATCAGCGTCATC
       ·  H  H  R  S  S  I  S  A  G  L  P  P  T  E  L  Q  L  L  D  A  Y  F  D  N  F  Q  P  F  S  P  L  L  D ·
892    ATCACCATCGTTCTTCAATTTCAGCTGGTCTACCACCTACAGAACTACAATTGCTAGATGCGTATTTTGACAACTTTCAACCTTTCTCACCACTACTAG
       ·  E  E  I  C  R  S  T  F  L  S  G  R  R  K  D  D  R  W  L  A  L  L  N  I  I  L  A  L  G  S  I  T  A ·
991    ATGAAGAAATCTGTCGCTCAACTTTTCTATCCGGCCGCAGAAAGGACGATCGCTGGTTAGCCCTACTCAATATAATCCTTGCACTAGGAAGCATCACCG
       ·  A  G  V  D  N  H  N  H  R  A  Y  F  E  R  S  M  S  F  L  N  L  K  T  L  G  N  P  S  L  E  V  V  Q ·
1090   CCGCAGGCGTGGATAACCACAACCACCGGGCATACTTTGAGCGTTCAATGAGCTTTCTCAATCTCAAAACCCTCGGCAACCCCAGTCTCGAAGTCGTCC
       ·  T  L  G  L  M  G  G  W  Y  C  H  Y  I  S  Q  P  N  L  G  Y  A  L  M  G  A  S  L  R  M  A  V  T  L ·
1189   AGACTCTGGGGCTCATGGGAGGCTGGTACTGCCACTACATCAGCCAGCCCAATCTCGGATACGCACTCATGGGCGCCTCGTTGCGTATGGCGGTTACAC
       ·  G  L  Q  R  E  P  P  F  D  S  H  S  L  G  G  N  T  A  R  S  G  Y  Q  E  F  K  R  R  V  W  W  S  L ·
1288   TAGGTCTGCAGCGAGAACCCCCGTTTGATAGTCATTCGTTGGGTGGCAATACTGCTAGATCAGGGTATCAGGAATTCAAACGGAGGGTTTGGTGGTCGC
       ·  C  C  L  E  T  W  G  H  E  T  L  G  R  P  S  M  D  F  F  G  P  S  I  T  V  K  F  P  H  L  L  D  K ·
1387   TTTGTTGTCTGGAGACATGGGGCCACGAGACACTGGGAAGGCCGAGTATGGATTTCTTTGGGCCGAGTATTACGGTCAAGTTTCCTCACTTACTTGATA
                                                                    E  N  Y  I  K  A  L  P  L  I  E  N  V  Q  F  I ·
1486   AGGTCCCTTCCCCCTTTCCAAAAGCTCCGAATTGAATATTAATGGGTAATAGGAGAATTACATCAAAGCCCTCCCTCTAATCGAAAACGTACAATTCAT
   · K  I  A  S  K  I  Q  E  S  L  A  A  L  P  T  L  T  H  T  E  L  L  N  L  D  S  Q  L  L  Q  W  W  N ·
1585   CAAAATCGCCTCCAAGATCCAGGAATCTCTCGCCGCACTCCCGACCCTAACACACACGGAACTGCTCAATCTAGATTCCCAACTTCTTCAATGGTGGAA
   ·     N  L  P  P  V  L  K  D  Y  S  P  C  P  D  A  L  Y  A  P  R  T  V  M  R  W  R  F  Y  N  Q  R  M  L ·
1684   TAACCTTCCACCAGTCCTGAAGGACTACTCCCCTTGCCCTGACGCTCTGTACGCCCCCGCGAACAGTAATGCGTTGGCGCTTCTACAATCAGCGCATGCT
       · L  Y  R  P  R  L  L  N  Y  A  M  R  R  I  P  L  I  A  I  K  D  E  E  R  T  A  V  Q  R  C  R  E  I ·
```

1783 CCTCTACCGCCCTCGCTTGCTGAATTATGCAATGCGCCGCATCCCCTTGATAGCAATCAAAGACGAAGAACGCACCGCAGTTCAACGATGTCGCGAAAT
· A Q V A I E D I S S T T A M N M N Q M I A W N A V W L V F Q A T M ·
1882 TGCACAGGTCGCGATTGAGGATATTTCCTCCACTACAGCGATGAATATGAATCAAATGATTGCGTGGAATGCGGTGTGGTTGGTGTTTCAGGCTACCAT
· V P L I Y L S A A A V V T D K D D G D G E V E A C K A Q V Q T A I ·
1981 GGTGCCGTTGATATATCTATCTGCTGCAGCAGTGGTAACTGATAAGGATGATGGCGATGGCGAGGTCGAAGCGTGTAAAGCGCAAGTCCAAACTGCGAT
· A T L D R M R R Y G H T A E R S L G M I S S I L E T I L H T P D T ·
2080 AGCGACGCTGGATCGTATGAGGCGGTATGGACATACGGCTGAGCGGTCGCTGGGGATGATCTCAAGTATTCTTGAGACTATTTTGCATACGCCTGACAC
· R L T T N A S A A N D Y E N T E T Q N Y P P I P T D Y Q P I T R E ·
2179 TAGATTGACGACAAATGCCTCGGCAGCGAATGACTATGAGAATACGGAAACCCAGAATTATCCTCCTATTCCGACGGATTATCAACCCATCACCCGAGA
· R V L D W T A T T A A T T G A T N T S F E N Y S S Q H M W E Y L S ·
2278 AAGGGTTTTGGATTGGACCGCTACTACCGCTGCGACTACCGGCGCCACCAATACGTCTTTTGAGAATTATTCGTCCCAGCATATGTGGGAGTATCTGAG
· W G E N N D I W A E L Y T S L N P Q E G A N F F D A R I Q *
2377 CTGGGGCGAGAATAATGATATCTGGGCTGAGCTGTATACTAGTTTGAATCCTCAAGAGGGGGCAAATTTCTTCGATGCTAGGATCCAGTGATCTTCGAT
2476 ATGCATGAAATGTATACTATCTTCTCTTACATGAGTCAATGTAGCACACTATTGACCTAAATAGCGATACTTCAATGGTGCATCACCCTCAGAAAGTCC
2575 CGTAACAATGACCACTTGCTCATCACCCGGCACAATGTCCATGGCACTATCACTAACTCGCACGCCATCCCTTTCCTCAAAAACGAGGCATTCACCGGC
2674 TTCCGTGCACTGAT

B

1 TGTTGCTTCTTGGCAGGCCCGAGTCACATTCAGTCTACTTGTTTTGTTTTGTTTCGTCTTATATACTCGTCTTATCTTGTCGAGTTACTTGAGGCAGTC
100 AATTAACTCACCGTTAGAGACTCGGCCGTCTCACCTTTCAACGAAAGGAATAGGTCGGTCTCCGGGACCCTTTCTGTCGCATCGACCGACCTAAGGCGG
199 TTGAAACGGCTCCACGCCATGTCTTCAGACTACCAATGACAATCCAGTGAAGTGGACTTCGGTTAAGAACATTTTTATAATCATTCGAAAGAGCATATT
M A Q P S Q T P G L D T L A E S S H Y V L E Q L R L A R E A ·
298 CGTTGCGAAAATGGCACAACCGTCGCAAACACCTGGTTTGGATACTCTCGCCGAGAGCTCGCATTACGTCCTGGAGCAATTGCGTCTTGCGCGCGAAGC
· D M N N S N N S N E F I K K D N K A A A D P V G S R M Q I M R S P ·
397 CGATATGAACAATAGCAATAACAGCAACGAATTTATCAAAAAAGATAACAAGGCCGCCGCGGATCCTGTTGGATCAAGAATGCAGATTATGCGCAGCCC
· L S D A R A G I R K H S A D T V A V R R R I S R A C D Q C N Q L R ·
496 GCTTTCGGATGCGAGGGCTGGTATTCGCAAGCATTCTGCTGATACGGTTGCGGTACGCCGGCGGATTAGTCGTGCTTGCGATCAGTGTAATCAACTACG
· T K C D G Q S P C A H C T ·
595 GACTAAATGTGATGGACAGAGTCCTTGTGCGCATTGTACCG*GTGAGATACATATCGCATACGAGTTTTATATAGAGGGAGCTAATTCATTGATTTTTTT*
· E R K S L S C E ·
694 *TTTTTTTTTTTTTTTTTTTTTTTTTTTTTTTTTTTCGGGACTGTATAGATTGTGGCCTGAGTTGCGAATATGCCAGAG*AAAGAAAGAGCCTGAGTTGCG
· Y A R E R K K R G K A S K K D I A E A A A K A A G G A R E S G T P ·
793 AATATGCCAGAGAAAGAAAGAAACGAGGAAAAGCATCGAAGAAAGATATTGCAGAAGCTGCTGCGAAGGCTGCTGGCGGTGCACGCGAATCTGGTACCC
· G Y D T V P D Q S S Q L S A A M A S E S E A R I N Q S R R S X S A ·
892 CGGGCTATGATACAGTCCCAGATCAGTCATCTCAATTATCGGCCGCGATGGCATCAGAATCCGAGGCCCGTATCAATCAATCGAGACGGTCATRTTCAG
· S Q V A D Q Q P G I A N L R E L A Q Q P P Q A R P R Q F F S S N M ·
991 CGTCGCAAGTCGCAGACCAACAGCCCGGTATTGCTAATCTGCGTGAACTTGCACAACAGCCGCCTCAAGCAAGGCCAAGGCAGTTTTTCTCGTCGAACA

· D S M T M N G Y G Q V Q N V D R S F I Q M P D L R D L Q P R S P S ·

1090 TGGATTCGATGACTATGAATGGATATGGACAGGTGCAGAATGTAGATCGATCATTCATACAGATGCCCGATTTGCGCGACTTGCAGCCGAGGTCACCGT

· A I V P V G L N G F H D A Y N M V D H A P T N I N Q Y Q Y P Q S G ·

1189 CTGCCATAGTTCCGGTTGGATTGAATGGATTCCACGATGCATATAACATGGTTGATCATGCGCCGACCAATATCAATCAGTATCAATATCCACAGTCTG

· E D T S T N H F P G L T P P V Q S P G W L P L P A P S V G F P S L ·

1288 GAGAAGATACATCGACAAACCACTTTCCTGGACTTACACCCCCTGTCCAGTCTCCCGGATGGTTACCTTTGCCAGCTCCATCTGTGGGCTTTCCATCAT

· N M S N G F T S T L K Y P V L E P L L P H I V S I I P Q S L A C D ·

1387 TGAATATGTCAAATGGCTTCACCAGCACATTGAAATATCCTGTGTTGGAGCCATTACTACCTCATATAGTCTCGATTATTCCACAATCTCTGGCATGTG

· L L D L Y F A S T S A S H I F P Q S P Y V V G H V F R K K S I L H ·

1486 ACCTTCTTGATCTTTACTTTGCCAGCACATCCGCCTCCCACATTTTTCCTCAGTCACCGTACGTGGTTGGACACGTATTCCGAAAGAAGTCTATCCTAC

· Q T Q P R T C S P A L L A S M L W V A A Q T S D A P F L T S P P S ·

1585 ATCAAACACAACCTCGCACATGCAGTCCCGCTCTATTGGCTAGCATGCTTTGGGTAGCAGCTCAGACTAGCGACGCGCCGTTTCTGACATCGCCTCCAT

· A R G R V C Q K L L E L T V G L L R P L I H G P T P G E T S P N Y ·

1684 CAGCACGGGGAAGGGTCTGTCAGAAGTTATTAGAGCTTACCGTTGGTTTACTCCGACCTTTGATCCATGGTCCCACGCCGGGAGAGACGTCACCTAACT

· A A N A V I N G V A L G G F G V S M D Q L G A Q S S A T G A V D D ·

1783 ATGCTGCTAATGCGGTTATTAATGGAGTTGCATTGGGCGGATTCGGTGTATCGATGGATCAACTAGGTGCCCAAAGCAGCGCTACCGGAGCAGTCGATG

· V A T Y I H L A T V V S A S E Y K A A S I R W W A A A W S L A R E ·

1882 ATGTTGCCACATACATTCATCTAGCGACTGTGGTATCAGCGAGCGAGTACAAAGCTGCAAGTATCCGCTGGTGGGCAGCAGCATGGTCTCTAGCACGCG

· L K L G R E L P P T P S Q P Q S H D R D G N V E M E P K A S R D T ·

1981 AACTAAAACTTGGACGTGAACTTCCCCCAACGCCTTCGCAGCCTCAAAGCCATGACCGAGATGGCAATGTCGAGATGGAGCCTAAAGCGTCGCGAGATA

· S H V T E E E R E E R R R I W W L L Y V M D R H L A L C Y N R P L ·

2080 CGAGCCACGTCACCGAGGAAGAACGCGAAGAGCGCAGACGTATATGGTGGCTTCTTTACGTGATGGATCGCCATCTAGCGTTATGCTACAACCGACCCT

· T L L D K E C E G L L Q P M N D D V W H T G D F A N A G Y R R A G ·

2179 TGACATTACTGGATAAAGAGTGTGAGGGTCTGCTACAACCGATGAACGACGACGTCTGGCATACCGGCGACTTTGCGAATGCCGGTTACCGACGAGCAG

· P S F E C T G H G M F G Y F L P L M T I L G E I V D L N H A R N H ·

2278 GTCCCAGCTTTGAATGTACCGGTCATGGAATGTTTGGGTATTTCTTACCATTGATGACTATTCTGGGTGAAATAGTCGATCTGAATCATGCGCGGAATC

· P R F G I H F R T S G E W D S H T V E I T R Q L D V Y E Q X L R E ·

2377 ATCCACGATTTGGCATCCACTTCCGAACGAGCGGCGAATGGGATAGTCATACTGTAGAAATTACTCGACAACTCGATGTTTACGAACAGAGMCTACGCG

· F E T R H X A S L G I G S E G A A D A G F N A A A P T G I D H V S ·

2476 AGTTTGAGACCAGACATRCGGCGTCTCTGGGTATTGGCAGTGAGGGCGCTGCTGATGCTGGCTTCAATGCTGCGGCGCCTACGGGAATTGATCATGTCA

· P S A R S S S T V G S R V N E S L M Q T K M V A A Y G T Y L M H V ·

2575 GTCCATCAGCAAGGTCGTCGAGCACAGTGGGGTCTCGCGTCAACGAGTCGCTAATGCAGACGAAAATGGTCGCCGCATATGGTACCTATCTCATGCACG

· L H I L L V G K W D P I S L L D D N D L W I S S E A F I T A M G H ·

2674 TCCTACACATCCTGCTTGTCGGAAAATGGGATCCCATCTCCTTATTGGATGACAACGATCTGTGGATCTCATCAGAAGCTTTTATTACAGCCATGGGCC

· A V K A A E A A S D I L E Y D P D L S F M P F F F G I Y L L Q G S ·

2773 ACGCCGTCAAGGCCGCCGAAGCAGCGTCCGATATCCTGGAATACGACCCCGATCTAAGTTTCATGCCGTTCTTCTTTGGCATTTATCTCTTACAAGGCA

· F L L L L T A D K L Q G D A D P S V V R A C E T I X R A H E A C V ·

2872 GTTTCCTGCTCTTACTGACCGCCGATAAATTGCAAGGCGACGCCGACCCCAGCGTTGTCCGGGCGTGCGAGACGATARTACGAGCCCATGAAGCATGTG

· V T L N T E Y Q R

2971 TTGTGACGCTGAATACGGAGTATCAG*GTTCGTTCCAATGTTTCAGCCTAATCCCCCCCTTTTTTTTCTGGTTAGATTGCTGACGAATATATTGCAG*CGC

N F R K V M R S A L A Q V R G R V P D D F G E Q Q Q R R R E V L A

3070 AATTTCCGTAAAGTCATGCGTTCCGCGCTGGCGCAAGTCCGTGGCCGTGTGCCGGATGATTTTGGCGAACAGCAGCAACGACGGCGNGAGGTACTTGCA

L Y R W T G D G S G L A L *

3169 CTTTATCGCTGGACAGGCGACGGCAGCGGATTAGCTTTGTGAGCGTTTCACGGTATATACATAGTATGTGATTTACGAGGTGTTGCATAAGGATGAGGC

3268 AGGATTGGGCAAAAGTTGGTTTCTTCAATATTGCTTTTTG

Figure S2. Nucleotide sequences for *clrB* (A) and *xlnR* (B) genes and corresponding amino acid sequences for ClrB (A) and XlnR (B) in the filamentous fungus *P. verruculosum*. Putative intron sequences are underlined. The zinc finger sequences are bolded in green boxes.

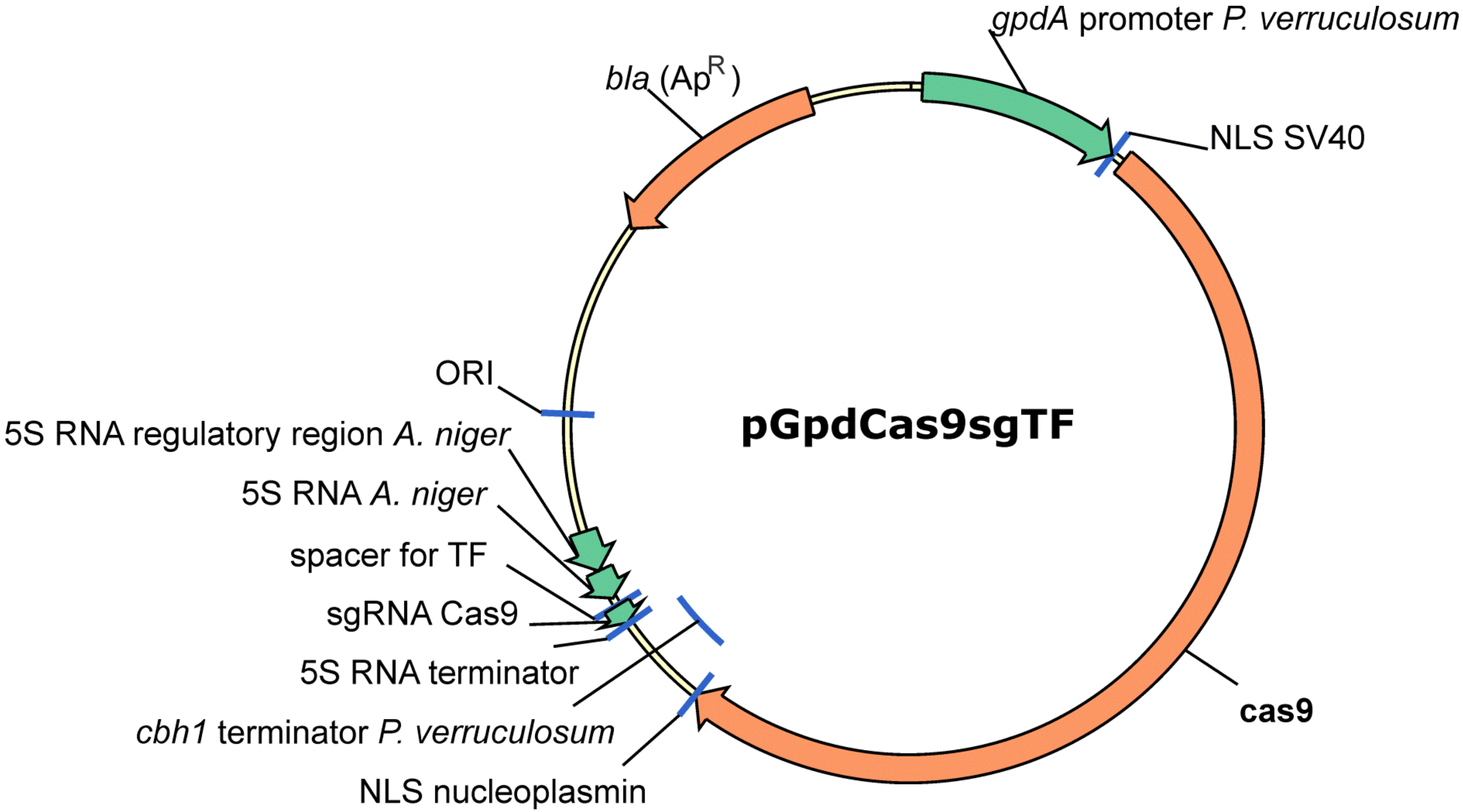

Figure S3. Scheme of the pGpdCas9sgTF plasmid. Major plasmid elements are indicated. NLS – nuclear localization signal. TF is gene of transcription factor (*clrB* or *xlnR*).

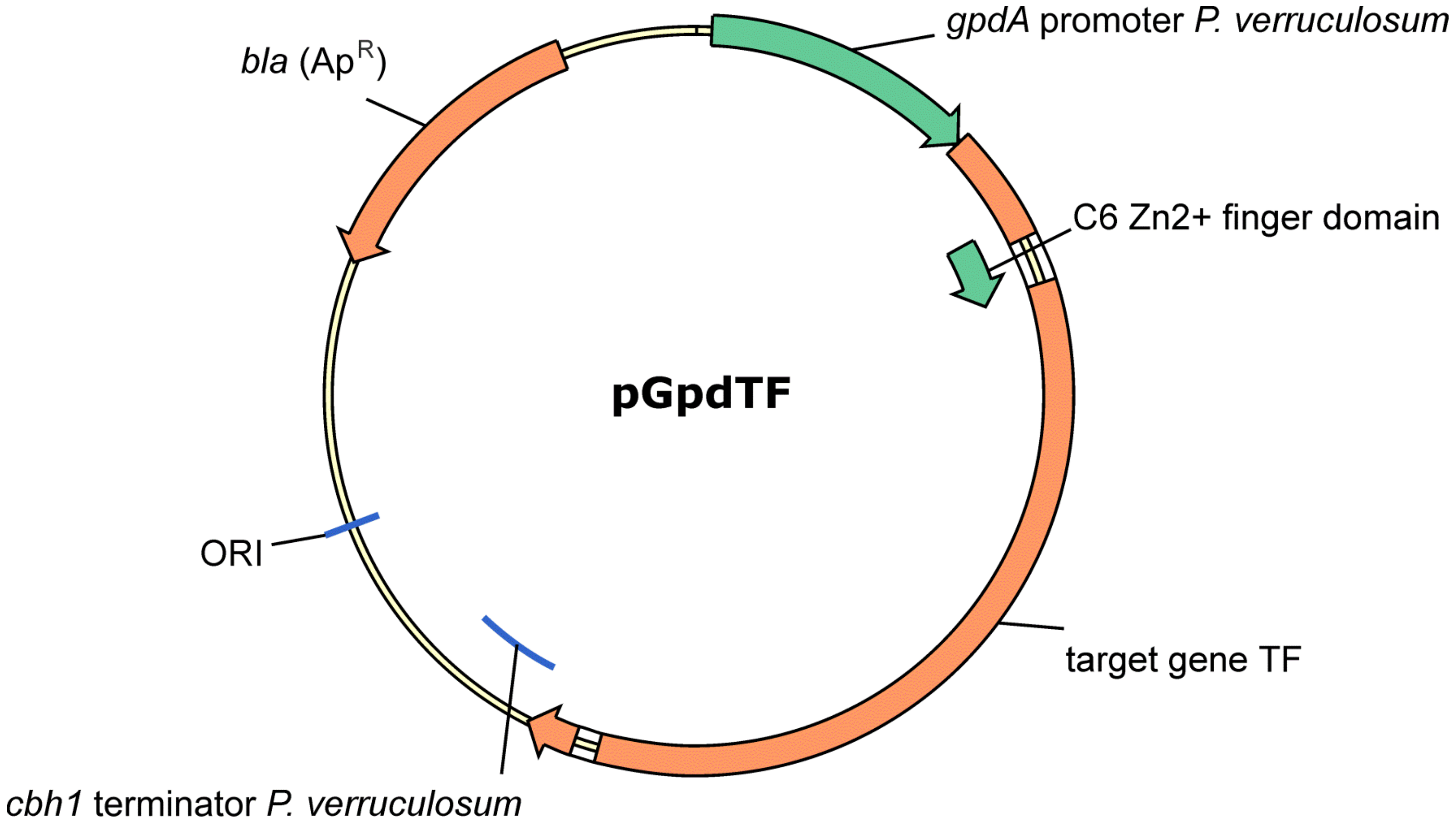

Figure S4. Scheme of the pGpdTF plasmid. Major plasmid elements are indicated. TF is gene of transcription factor (*clrB* or *xlnR*).
